# Leveraging Social Networking Sites for an Autoimmune Hepatitis Genetic Repository: Pilot Study to Evaluate Feasibility

**DOI:** 10.2196/jmir.7683

**Published:** 2018-01-18

**Authors:** Megan Comerford, Rachel Fogel, James Robert Bailey, Prianka Chilukuri, Naga Chalasani, Craig Steven Lammert

**Affiliations:** ^1^ Division of Digestive and Liver Diseases Indiana University School of Medicine Indianapolis, IN United States; ^2^ Department of Medicine Indiana University School of Medicine Indianapolis, IN United States

**Keywords:** autoimmune hepatitis, social media, rare disease

## Abstract

**Background:**

Conventional approaches to participant recruitment are often inadequate in rare disease investigation. Social networking sites such as Facebook may provide a vehicle to circumvent common research limitations and pitfalls. We report our preliminary experience with Facebook-based methodology for participant recruitment and participation into an ongoing study of autoimmune hepatitis (AIH).

**Objective:**

The goal of our research was to conduct a pilot study to assess whether a Facebook-based methodology is capable of recruiting geographically widespread participants into AIH patient-oriented research and obtaining quality phenotypic data.

**Methods:**

We established a Facebook community, the Autoimmune Hepatitis Research Network (AHRN), in 2014 to provide a secure and reputable distillation of current literature and AIH research opportunities. Quarterly advertisements for our ongoing observational AIH study were posted on the AHRN over 2 years. Interested and self-reported AIH participants were subsequently enrolled after review of study materials and completion of an informed consent by our study coordinator. Participants returned completed study materials, including epidemiologic questionnaires and genetic material, to our facility via mail. Outside medical records were obtained and reviewed by a study physician.

**Results:**

We successfully obtained all study materials from 29 participants with self-reported AIH within 2 years from 20 different states. Liver biopsy results were available for 90% (26/29) of participants, of which 81% (21/29) had findings consistent with AIH, 15% (4/29) were suggestive of AIH with features of primary biliary cholangitis (PBC), and 4% (1/29) had PBC alone. A total of 83% (24/29) had at least 2 of 3 proposed criteria: positive autoimmune markers, consistent histologic findings of AIH on liver biopsy, and reported treatment with immunosuppressant medications. Self-reported and physician records were discrepant for immunosuppressant medications or for AIH/PBC diagnoses in 4 patients.

**Conclusions:**

Facebook can be an effective ancillary tool for facilitating patient-oriented research in rare diseases. A social media-based approach transcends established limitations in rare disease research and can further develop research communities.

## Introduction

Challenges in recruiting patients into rare disease clinical studies contribute to wide knowledge gaps in understanding disease pathogenesis, natural history, and optimal therapeutic approaches. In the United States, rare diseases are defined as affecting fewer than 200,000 people at a given time. Despite these seemingly small numbers, there are close to 7000 different rare diseases that impact more than 25 million Americans (7% of the population) [1]. Autoimmune hepatitis (AIH), a chronic liver disease with a prevalence of 24 in 100,000 people [2], is characterized by immune-mediated destruction of hepatic parenchyma, which can result in cirrhosis and death [3-6]. High-impact studies in this field have historically required coordinated multicenter approaches to achieve the participant numbers that provide meaningful results. Obstacles encountered with such studies include increased cost and effort to locate collaborators, successfully train study personnel, and obtain additional ethics approvals [7,8]. Time and money spent on travel by participants can also be barriers to recruitment even for single-site studies. Even when these coordinated approaches are successful, the low density of academic research centers and sparse distribution of patients in North America leaves a large percentage of patients without access to research opportunities to take a more active role in scientific advancement of their own disease [7,9].

The digital age has created new tools for rare disease research, as many proactive patients connect with each other and investigators through online support groups on social networking sites (SNS) such as Facebook and Twitter [10]. With nearly 2 billion daily active users, Facebook is the largest SNS worldwide and consumes 40 minutes of the average American user’s day [11,12]. Individuals in disease-specific groups are motivated to stay up to date with the latest research relevant to their conditions and have even engaged investigators to initiate studies within their respective online groups [13]. Collaboration between such active SNS patient groups and technologically skilled investigators can potentially overcome critical challenges in rare disease research and reduce the need for multicenter approaches.

Considering the established barriers to AIH research and the abundance of AIH patient support groups on SNS, we formed the physician-led Autoimmune Hepatitis Research Network (AHRN) Facebook group to foster patient support, share current AIH information, and promote AIH research efforts. In 2014, we initiated an observational study of patients with AIH (Genetic Repository of Autoimmune Liver Diseases and Contributing Exposures, GRACE Study) for investigating genetic and environmental underpinnings in disease development and clinical outcomes. We conducted a pilot study to assess whether a Facebook-based methodology is capable of recruiting geographically widespread participants into AIH patient-oriented research and obtaining quality phenotypic data.

## Methods

### Autoimmune Hepatitis Research Network

We established the AHRN on Facebook in 2014 to provide an online repository of summarized AIH literature, discussion of study results and application to patient care, and opportunities to participate in our ongoing AIH research studies. As of May 2017, the AHRN Facebook group has 1640 members and is led and moderated by a hepatologist (CL) from Indiana University (IU). New membership has grown by individuals finding the page via Facebook search results, referrals shared in other AIH Facebook groups by our members, and direct physician-to-clinical-patient marketing. The AHRN is a private Facebook group; individual users wishing to join must be granted approval from the group moderator. Once approved, members are able to view and share content with other group members. A private group setting has the benefit of protecting the privacy of members by hiding their group affiliation from nonmember friends and allows group moderators to screen potential members for illegitimate accounts.

### Study Recruitment

We posted a GRACE study recruitment advertisement, consisting of a recruitment message, study summary sheet, and representative study image, on the AHRN Facebook group after obtaining institutional review board approval. The study summary sheet outlined study aims, procedures, inclusion criteria, and the study coordinator’s contact information (phone and email). Inclusion criteria consisted of individuals 18 years and older, diagnosis of AIH or AIH with features of overlap disease (primary biliary cholangitis [PBC] or primary sclerosing cholangitis [PSC]) established by a medical doctor, and no provision of medical care at IU or surrounding affiliate institutions. The study advertisement was posted on the AHRN Facebook group at quarterly intervals over 24 months by the group moderator. The study advertisement directed interested individuals to contact the study coordinator to discuss study objectives and subsequently give consent by phone.

After verbal consent, a study packet containing epidemiologic surveys [14,15], an Oragene DNA saliva kit (DNA Genotek Inc), study instructions, consent documents, health information release form, and prepaid return envelope were mailed to the participant’s home address. Self-reported demographic parameters and medical history were collected from a single survey [14]. Participants were then assigned a study identification number linking all completed materials. Upon receipt of study materials, the participant’s health information release was faxed to the physician treating the AIH in order to collect supportive data related to diagnostic testing. Requested medical records included procedure notes and reports of liver biopsy and pathology and antinuclear antibody, antismooth muscle antibody, antiliver kidney microsomal antibody, antisoluble liver antigen, antimitochondrial antibody (AMA), both cytoplasmic and perinuclear antineutrophil cytoplasmic, immunoglobulin G, and viral hepatitis serologies. Failure to receive a participant’s study materials or outside medical records within 1 month of distribution resulted in repeat contact to each entity up to 3 times. Outside medical record nonresponse was further targeted with a follow-up phone call to the appropriate clerk on the day of medical release transmission.

### Data Collection and Analysis

Once received, outside medical records were reviewed and tabulated into the local AIH database by study personnel. Missing clinical and laboratory support data for AIH diagnosis from outside records were identified and subsequently targeted with repeat release of information requests. Collected results (including saliva kit, epidemiologic surveys, and outside diagnostic reports) were stored in a secure location.

### DNA Collection and Quantification

Saliva was collected from the subjects using an Oragene collection kit and stored according to kit instructions. DNA was isolated from 500 µL of saliva in PrepIT-L2P Purifier Reagent (Lot PT150219-A, DNA Genotek Inc), and remaining collected saliva sample was saved for future study.

## Results

### Recruitment Outcomes

We enrolled 29 participants in 2 years between June 2014 and June 2016. Participants were from 20 different states, with 27 (93%) indicating a primary residence outside of Indiana and a median distance of 727 miles from our center. Of 29 participants, 28 (97%) successfully completed and returned study instruments within 3 months of the coordinator-led phone consent. All participants were female, 90% (26/29) were white, and median age at enrollment was 52 years.

### Participant Characteristics

Of 28 participants, 20 (71%) subjectively reported a diagnosis of AIH alone, while 29% (8/28) reported AIH with features of PBC (Table 1). Among all included participants, 46% (13/28) reported concurrent, extrahepatic autoimmune disease (data not shown). Other frequent medical conditions and symptoms reported included history of contraceptive use (26/28, 93%), history of multiple urinary tract infections (19/28, 68%), frequent fatigue (19/28, 68%), occasional to constant itching (13/28, 46%), and right upper abdominal discomfort (17/28, 61%).

All participants recorded their current medication list within an epidemiologic survey provided at enrollment (Table 2). Discrepancies were found between the participant surveys and medical records for immunosuppressant medication usage. Additionally, 3 participants reported no active immunosuppressant use, which was contradicted by their outside medical records. One participant had no documented evidence of immunosuppression in the physician notes and also had an incomplete simplified score, high AMA titer, and a biopsy consistent with PBC alone.

Appropriate outside medical records were received after the first request along with the medical information release form for 79% (23/29) of participants (Table 1). A total of 50% (13/26) of available liver biopsies were typical for AIH, 1 (4%) indicated PBC only, and 12 (46%) were identified as compatible with AIH according to the simplified criteria histologic findings [16]. Of the participants with definite AIH per the simplified criteria, 25% (1/4) had a clinically reported a positive AMA titer as well as findings on biopsy of AIH with features of PBC.

Three-quarters of the participants (22/29, 76%) had available autoimmune markers for the simplified score assessment. However, there were a number of missing values for the simplified criteria given the nature of data collection. Therefore, in order to enroll AIH patients into the GRACE study, we sought to categorize the participants most likely to have AIH based on meeting at least 2 of 3 lab- or clinician-reported criteria: positive autoimmune markers (simplified criteria), liver biopsy with typical findings of AIH, and/or reported treatment with immunosuppressant medications. Of 29 participants, 24 (83%) met at least 2 of 3 criteria, with 19 of these participants (79%) satisfying all 3.

### DNA Isolation Results

Saliva samples were obtained from all 29 study participants. All collected samples were at least 2 mL in volume. DNA isolation from representative saliva samples yielded sufficient quantity and quality of DNA for genetic investigations. Median value of DNA yield was 41 µg per 500 µL saliva sample (interquartile range [IQR] 41.86) and 260/280 ratio was 1.84 (IQR 0.125).

**Table 1:** Objective participant data collected from outside medical records.

Study no.	No. of medical record attempts	Simplified AIH^a^ score parameters					Immunosuppression per MD^c^ note	Diagnosis per biopsy	
		Autoantibody score	IgG^b^ score	Viral score	Histologic score	Complete simplified score			
1	1	0	2	2	2	6	Yes		AIH
2	3	2	—	2	0	—	No		PBC^d^
3	3	2	—	—	2	—	Yes		AIH
4	3	—	—	2	2	—	Yes		AIH
5	1	2	2	2	2	8	Yes		AIH
6	3	2	—	—	2	—	Yes		AIH
7^e^	3	—	—	—	—	—	Yes		—
8	1	2	—	2	1	—	Yes		AIH
9	2	2	2	2	2	8	Yes		AIH
10	1	2	0	2	2	6	Yes		AIH/PBC
11	3	—	—	—	2	—	Yes		AIH
12	3	—	—	—	1	—	Yes		AIH
13	3	0	—	2	1	—	Yes		AIH
14	1	2	2	2	2	8	Yes		AIH/PBC
15	2	2	0	2	2	6	Yes		AIH
16	1	—	—	0	1	—	—		AIH
17	1	0	0	2	1	3	Yes		AIH
18	1	1	2	2	1	6	Yes		AIH/PBC
19	1	2	0	2	2	6	Yes		AIH
20	1	—	—	2	2	—	Yes		AIH/PBC
21	1	2	0	2	1	5	Yes		AIH
22	1	2	1	2	2	7	Yes		AIH/PBC
23	1	2	—	—	2	—	Yes		AIH
24^e^	1	—	—	—	—	—	—		AIH
25	1	0	0	0	1	1	Yes		AIH
26	1	2	2	2	2	8	Yes		AIH
27	1	2	0	2	2	6	Yes		AIH or AIH/PBC
28	1	1	—	2	1	—	Yes		AIH
29	1	2	—	2	1	—	Yes		AIH

^a^AIH: autoimmune hepatitis.

^b^IgG: immune globulin.

^c^MD: medical doctor.

^d^PBC: primary biliary cholangitis.

^e^Participants without biopsy results available for review.

**Table 2:** Subjective participant data collected from epidemiologic survey.

Study no.	AIH^a^	PBC^b^	No. of immuno-suppression prescriptions	UDCA^c^
1	Yes	Yes	2	No
2	Yes	Yes	0	Yes
3	Yes	No	0	Yes
4	Yes	No	2	Yes
5	Yes	No	2	Yes
6	Yes	No	2	No
7	Yes	Yes	2	No
8	Yes	No	1	No
9	Yes	No	1	No
10	Yes	Yes	1	Yes
11	Yes	No	2	No
12	Yes	No	0	No
13	Yes	No	0	No
14	Yes	Yes	0	Yes
15	Yes	No	1	Yes
16	Yes	No	0	No
17	Yes	No	1	No
18	Yes	Yes	1	Yes
19	Yes	No	1	Yes
20	Yes	Yes	—	Yes
21	Yes	No	0	No
22	Yes	Yes	1	Yes
23	Yes	No	2	No
24	Yes	No	2	No
25	Yes	No	2	No
26	Yes	No	2	No
27	Yes	Yes	2	No
28	Yes	No	1	No
29	Yes	No	1	No

^a^AIH: autoimmune hepatitis.

^b^PBC: primary biliary cholangitis.

^c^UDCA: ursodeoxycholic acid.

## Discussion

### Principal Results

Sustained AIH research advances are continually impeded by low participant enrollment numbers, limited and disconnected investigators, and poor engagement of geographically spread patients wanting to contribute to scientific progress [9]. However, social media presents a unique platform to address these significant investigational barriers. Our research engagement with the AHRN Facebook group supports patient-oriented research and facilitates participation in research studies even from a distance. Furthermore, we assert that our research team correctly phenotyped and recruited 24 AIH patients and 4 AIH patients with overlap features of PBC to the GRACE study from outside our institutional reach.

The retrieval of supporting outside records and eventual clinical diagnosis of AIH can be challenging for participants recruited both locally and remotely. Remote access was impeded most frequently by delayed response from corresponding medical record departments. Despite the obstacles inherently imposed by the study design, we were able to successfully collect epidemiologic questionnaires and biologic samples from all 29 participants using Facebook. While the effort spent on retrieval of outside records was time intensive, the passive approach to recruitment required less time spent identifying and recruiting patients enrolled locally through traditional methods. The successful recruitment of 29 subjects was substantial for the GRACE study, as our traditional approach at a large academic center provided only 120 participants (24% more cases obtained with social media) in the same duration of study.

SNS have evolved rapidly over the past decade to support our need for social connectedness and instant digital knowledge. Application of these tools in rare disease research is important, as they transcend well-established limitations such as cost, prolonged study courses, and geographic barriers. Our study was cost- and time-efficient and effective at recruiting patients up to 2200 miles away from our medical center. Furthermore, the cohort was similar to prior work according to gender (female predominant), ethnicity, concurrent autoimmune illnesses, fatigue, and itch [2,17]. Participants in similar studies at our institution are often reimbursed US $25 for expenses related to travel, parking, or time burden. Those expenses were not incurred by the GRACE study participants and thus reduced overall cost of study conduct by approximately US $725. Furthermore, our approach to research also reduces time burden for study participants. Our Web-based approach allows participants to complete the study procedures at their convenience rather than on the schedule of the study team and eliminates the additional time spent traveling to and from the study center.

### Limitations

In our study, prospective participants quickly engaged our study coordinator for recruitment. However, self-attestation of AIH diagnosis and current treatment strategies were not always congruent with their physician-composed medical records. Furthermore, we found certain medical records had insufficient documentation or lacked specific workup components. Our assessment of at least 2 of 3 key criteria, as outlined above, suggests AIH diagnosis in 24 out of 29 study participants (83%). Other studies have shown similar levels of agreement between self-reported diagnosis data and medical record documentation using traditional research methods, suggesting this challenge is not unique to social media–based research [18,19].

Successful implementation of a social media–based methodology comes with its own unique set of challenges. While SNS can supplement traditional recruitment, the process of sending and receiving medical records and study materials from affiliated institutions and participants proved to be time-intensive for the study team. Nonetheless, the time cost associated with this methodology is considerably less compared to the effort required to initiate recruitment at multiple study sites to achieve a similar geographical reach. Additionally, the demographics of AIH patients support a social media approach as they align well with the SNS user demographics [20]. This group may be better able to provide accurate self-reported data, as it has been found that female gender and higher educational attainment, characteristics of SNS users, are associated with increased uniformity between patient reports and medical records [19]. The demographic similarity between AIH patients and SNS users may not exist for some rare diseases and must be considered when evaluating the applicability of social media methods to specific patient populations.

Finally, a notable challenge of implementing SNS in medical research is simply its acceptability by providers and health systems. The medical community has been hesitant to embrace the potential of this approach to research recruitment, in part due to the risk of Health Insurance Portability and Accountability Act violations and the potential for demographic biases [9,21]. Furthermore, low acceptance may stem from slow adoption of new technologies, particularly in fast-paced clinical environments, and research regarding the use of SNS in medical research is still limited [22]. The rise of mobile health technologies using mobile phones and wearable devices will create new opportunities for conducting remote clinical studies drawing participants from SNS. We believe as these technologies become more prevalent in clinical and research settings, acceptance of new research methodologies such as SNS will also increase. Failure to integrate this model represents a missed opportunity, as many of the over 2 billion daily Facebook users have actively sought medical information or participated in health-related digital support groups [23,24].

### Future Applications and Conclusions

We foresee opportunities for social media to help connect like-minded investigators for research collaborations [25]. As we continue to supplement site-based recruitment for GRACE with Facebook-based participant enrollment, we anticipate additional opportunities for social media to be used to complement traditional studies through study advertisements, recruitment opportunities, collaborations with patient groups, or study-specific groups or pages designed for easy communication with study participants. Using SNS for recruiting participants for research regarding rare diseases such as AIH serves as a viable bridge to connect patients to investigators despite geographical barriers and a lack of local opportunities. We are confident our method could become a frontrunner in the improvement of low study numbers in rare disease research. In overcoming recruitment limitations, we now seek a way to efficiently expedite the time between recruiting individuals, obtaining their biologic samples and epidemiological data, and confirming their diagnosis.

In summary, SNS can be an effective tool for facilitating patient-oriented research in AIH. We have successfully implemented a Facebook-based methodology to engage individuals unable to participate in research through traditional methods due to geographic or financial barriers. Mindful use of SNS can transcend established limitations in rare disease research and further cultivate powerful and engaged research communities.

## Abbreviations

AHRN: Autoimmune Hepatitis Research Network
AIH: autoimmune hepatitis
AMA: antimitochondrial antibody
GRACE: Genetic Repository of Autoimmune Liver Diseases and Contributing Exposures
IQR: interquartile range
IU: Indiana University
PBC: primary biliary cholangitis
PSC: primary sclerosing cholangitis
SNS: social networking sites
UDCA: ursodeoxycholic acid
